# Novel Therapeutics in Soft Tissue Sarcoma

**DOI:** 10.3390/cancers17010010

**Published:** 2024-12-24

**Authors:** Leonidas Mavroeidis, Andrea Napolitano, Paul Huang, Robin L. Jones

**Affiliations:** Sarcoma Unit, The Royal Marsden Hospital and Institute of Cancer Research, London SW3 6JZ, UK

**Keywords:** soft tissue sarcoma, therapeutics, drug development, treatment

## Abstract

There has been noteworthy progress in molecular characterisation and therapeutics in soft tissue sarcomas. Novel agents have gained regulatory approval by the FDA. Examples are the tyrosine kinase inhibitors avapritinib and ripretinib in gastrointestinal stromal tumours (GIST), the immune check point inhibitor atezolizumab in alveolar soft part tissue sarcoma, the γ-secretase inhibitor nirogacestat in desmoid tumours, the NTRK inhibitors larotrectinib and entrectinib in tumours with *NTRK* fusions, the mTOR inhibitor nab-sirolimus in PEComa, and the EZH-2 inhibitor tazemetostat in epithelioid sarcoma. The FDA has also recently granted accelerated approval for autologous T-cell therapy with afami-cel in patients with HLA-A*02 and MAGE-A4-expressing synovial sarcoma. There are other promising treatments that are still investigational, such as MDM2 and CDK4/6 inhibitors in well-/dedifferentiated liposarcoma, immune checkpoint inhibitors in the head and neck angiosarcoma and a subset of patients with undifferentiated pleomorphic sarcoma, and PARP inhibitors in leiomyosarcoma. The challenges in drug development in soft tissue sarcoma are due to the rarity and the molecular heterogeneity of the disease and the fact that many subtypes are associated with complex karyotypes or non-targetable molecular alterations. We believe that progress maybe possible with a better understanding of the complex biology, the development of novel compounds for difficult targets such as proteolysis targeting chimeras (Protacs), the utilisation of modern clinical trial designs, and enhanced collaboration of academia with industry to develop treatments with a strong biologic rationale.

## Simple Summary

Soft tissue sarcomas comprise a diverse group of tumours. Their rarity and heterogeneity make the development of novel therapeutics challenging while the knowledge in this topic is fragmented. Here, we provide a comprehensive review on drugs that have recently gained regulatory approval and the most significant advances in investigational therapeutics. We give emphasis on the underlying biology that guide the development of these treatments and we provide our perspective for future progress.

## 1. Introduction

Soft tissue sarcomas (STS) constitute a group of rare tumours of mesenchymal origin accounting for approximately 1% of adult malignancies, comprised of more than 150 different histologic subtypes [1]. The rarity, histologic heterogeneity, and diversity in anatomic distribution necessitates management in high-volume, specialised centres with available expertise. The clinical outcome has been shown to be better when the treatment is guided by a multidisciplinary team [2].

Genetically, STS are characterised by four types of molecular alterations, either driver somatic mutations, for example, mutations in the *KIT* gene in GIST [3], or chromosomal translocations, which result in fusion genes such as the SS18-SSX gene fusion due to the t(X;18) (p11.2;q11.2) translocation in synovial sarcoma [4], or neochromosomes, as in the case of well-differentiated and dedifferentiated liposarcoma due to amplification of the chromosomal 12q13-15 region [5], or complex karyotypes like in the case of leiomyosarcoma [6].

For localised disease, surgery is the mainstay of treatment. Multimodality treatment with chemotherapy and radiotherapy in addition to surgical resection may be incorporated depending on the anatomic location, size, grade, and histologic subtype [7].

In the advanced disease setting, chemotherapy remains the cornerstone of treatment. Doxorubicin is the preferred first-line either alone or in combination with another chemotherapeutic for most histologic subtypes [8]. It is unclear whether combination chemotherapy improves overall survival (OS), but it can increase the response rate and progression-free survival (PFS); thus, it may [9] be used when maximisation of response is required, for instance, in cases of borderline resectable disease or impending organ dysfunction [10]. Ifosfamide can be added in patients with synovial sarcoma [11,12] and dacarbazine in patients with leiomyosarcoma [13]. Patients with contraindications to anthracyclines may be offered gemcitabine as a single agent or in combination with docetaxel [14] or dacarbazine [15]. Pazopanib is also an option for patients with non-adipocytic soft-tissue sarcoma who have previously progressed to chemotherapy [16]. Trabectedin can also be offered as a second-line treatment [17,18]. Eribulin can be used as a later-line treatment for patients with liposarcoma [19].

The use of targeted therapy in STS is limited to only a few subtypes at present. Tyrosine kinase inhibitors are the standard treatment for gastrointestinal stromal tumours (GISTs) with oncogenic mutations in the KIT protooncogene (*KIT*) and the Platelet-Derived Growth Factor Receptor Alpha (*PDGFRA*) genes [20]. Imatinib is effective in patients with dermatofibrosarcoma protuberans, given that the majority of patients carry the *COL1A1::PDGFB* gene fusion due to t(17;22) translocation [21]. Crizotinib has shown efficacy in patients with inflammatory myofibroblastic tumours with *ALK* gene fusions [22]. Larotrectinib [23] and entrectinib [24] are effective when *NTRK* gene fusions are present, such as in infantile fibrosarcoma, a subset of patients with inflammatory myofibroblastic tumours, extra rare cases of GIST, or non-specified STS. Hormonal therapy in uterine sarcoma with estrogen receptor (ER) and progesterone receptor (PR) expression is another targeted treatment option [25].

Olaratumab, a monoclonal antibody that specifically binds PDGFRα, was shown to improve survival in combination with doxorubicin as compared to doxorubicin alone in patients with advanced STS in an open-label Phase 1b, randomised, Phase 2 study [26]. This led to accelerated regulatory approval in several countries. However, the confirmatory ANNOUNCE Phase 3 trial failed to confirm this survival benefit, which highlights the importance of validation in confirmatory Phase 3 trials [9].

Overall, immune checkpoint inhibitors (ICIs) have shown modest activity in all-comers clinical trials in STS [27,28]. However, there is notable efficacy in certain histologies. The anti-PDL1 atezolizumab is effective in patients with alveolar soft part tissue sarcoma [29], while other ICIs have shown promising efficacy in a subset of patients with undifferentiated pleomorphic sarcoma (UPS) [30] and angiosarcoma, especially in cutaneous angiosarcoma of the head and neck [31,32].

In this review, we will discuss the most recent advances in approved and investigational therapeutics in soft-tissue sarcoma in relation to the underlying biology.

## 2. Gastrointestinal Stromal Tumours (GIST)

Gastrointestinal stromal tumours (GIST) represent the most common STS of the gastrointestinal tract. They likely arise from the interstitial cells of Cajal (ICCs) and are characterised by overexpression of the tyrosine kinase receptor KIT (CD117) on immunohistochemistry (IHC). Approximately 70–80% of the cases carry a mutation in the *KIT* gene and 5–10% in the *PDGFRA* gene, while the rest lack mutations in either of these genes and belong to the wild-type GIST category. The succinate dehydrogenase (SDH)-deficient GIST represents a subset of wild-type GIST characterised either by a loss-of-function mutation in one of the *SDH* genes or epigenetic silencing of the *SDHC* gene. The remaining wild-type GIST harbour mutations in the Neurofibromatosis 1 (*NF1*) gene or carry the *BRAFV600E* mutation or an *NTRK*-fusion [3,33].

Imatinib is the first line, while sunitinib and regorafenib constitute subsequent lines of treatment in patients with advanced GIST with sensitive *KIT* and *PDGFRA* mutations. Recently, ripretinib was approved as a later-line treatment, while avapritinib was approved for the insensitive *PDGFRA* (D842V) mutated GIST [33].

In particular, ripretinib was approved by the US Food and Drug Administration (FDA) for patients with advanced GIST who have received three or more TKIs based on the results of the Phase III trial INVICTUS. In this study, patients with advanced GIST intolerant or refractory to imatinib, sunitinib, and regorafenib were randomised to ripretinib or placebo. Ripretinib led to improvement in the median overall survival (mOS) (15.1 versus 6.6 months, hazard ratio [HR]: 0.36, 95% CI, 0.20–0.62; nominal *p* = 0.0004) and the median progression-free survival (mPFS) (6.3 versus 1.0 months, HR: 0.15, 95% CI, 0.09–0.25; *p* < 0.0001). The safety profile was acceptable. The most common adverse reactions (≥20%) were alopecia, fatigue, nausea, abdominal pain, constipation, myalgia, diarrhoea, decreased appetite, palmar-plantar erythrodysesthesia (PPE), and vomiting. Primary cutaneous malignancies, hypertension, and cardiac dysfunction were also reported as toxicities [34]. In addition, these results have been verified by real-world experience [35].

Ripretinib was also evaluated as a second-line treatment in the INTRIGUE trial but without showing superiority to sunitinib. In this randomised, open-label, Phase III study, patients with advanced GIST who had previously progressed or were intolerant to imatinib were randomised to ripretinib or sunitinib. However, ripretinib did not demonstrate significant improvement over sunitinib in mPFS either in the overall intention to treat population (ITT) (8.0 versus 8.3 months, HR: 1.05, 95% CI, 0.82–1.33; nominal *p* = 0.72) or the ITT population with *KIT* exon 11 mutation (8.3 versus 7.0 months, HR: 0.88, 95% CI, 0.66–1.16; *p* = 0.36) but was associated with a favourable toxicity profile [36].

Interestingly, an exploratory analysis of this trial revealed two mutually exclusive populations with differential treatment effects based on the profile of secondary-resistance *KIT* mutations detected in circulating tumour DNA(ctDNA) [37]. Patients with primary *KIT* exon 11 mutation and secondary-resistance mutations in the adenosine triphosphate (ATP)-binding pocket (exons 13/14) had improved mPFS with sunitinib compared with ripretinib (15.0 versus 4.0 months, HR: 3.94, 95% CI, 1.71–9.11). Conversely, patients with primary *KIT* exon 11 mutation and secondary-resistance mutations in the activation-loop (exons 17/18) had improved mPFS with ripretinib compared with sunitinib (14.2 versus 1.5 months, HR: 0.22, 95% CI, 0.11–0.44). Patients with only the baseline *KIT* 11 mutation had better mPFS with sunitinib compared with ripretinib (16.3 versus 2.2 months, HR: 2.24, 95% CI, 0.99–5.09), whereas no difference was detected in mPFS between sunitinib and ripretinib in patients with concomitant mutations in both the ATP-binding pocket and activation loop of *KIT* (HR: 1.07, 95% CI, 0.41–2.84) [37]. The superiority of ripretinib to sunitinib in patients with secondary mutations in exons 17/18 of the *KIT* gene justified the conduct of the INSIGHT trial, a randomised, open-label, Phase III study of ripretinib versus sunitinib in patients with advanced GIST previously treated with imatinib with co-occurring *KIT* mutations in exon 11 and exon 17 and/or 18 confirmed by ctDNA analysis [38].

Avapritinib was shown to have unprecedented efficacy in patients with resistant to imatinib D842V mutation in exon 18 of the *PDGFRA* gene in the Navigator trial. This was a two-part, open-label, dose-escalation and dose-expansion Phase I study. In total, 56 patients with unresectable GIST with *PDGFRA* D842V mutation were treated with avapritinib [39]. In an updated analysis, the ORR was 91% (51/56 patients), the duration of response (DOR) was 27.6 months (95% CI, 17.6—not reached [NR]), and the mPFS was 34.0 months (95% CI, 22.9-NR) [40]. The most frequent adverse events were nausea (68%), diarrhoea (66%), and cognitive effects, which encompassed memory impairment, confusional state, cognitive disorder, and encephalopathy (57%). These results led to the FDA approval of avapritinib for patients with unresectable or metastatic GISTs with a *PDGFRA* exon 18 mutation.

However, avapritinib failed to show superiority to regorafenib as a third- or later- line treatment in molecularly unselected GIST in the VOYAGER trial. In this Phase III study, patients with unresectable or metastatic GIST were randomised to 1:1 to avapritinib or regorafenib. Crossover was permitted upon confirmation of disease progression (PD) by central review. No difference in median PFS between avapritinib and regorafenib (4.2 versus 5.6 months, HR: 1.25, 95% CI, 0.99–1.57; *p* = 0.055) was observed, and the treatment-related adverse events (TRAEs) (any grade or grade ≥ 3) were similar for avapritinib and regorafenib.

Given the role of the chaperone heat shock protein 90 (HSP90) in stabilising the KIT and PDGFRA proteins, the HSP90 inhibitor pimitespib was also evaluated in a randomised Phase III trial [41]. The CHAPTER-GIST-30 study showed improvement of pimitespib over placebo in mPFS (2.8 versus 1.4 months, HR: 0.51, 95% CI, 0.30–0.87; one-sided *p* = 0.006) and in the cross-over-adjusted mOS (13.8 versus 7.6 months, HR: 0.42, 95% CI, 0.21–0.85; one-sided *p* = 0.007) in patients with advanced GIST refractory to standard TKIs. The most common TRAEs were diarrhoea (74.1%) and decreased appetite (31.0%). Grade 1 night blindness occurred in 13.8% of patients [41]. Pimitespib gained regulatory approval in Japan.

Clinical trials with novel TKIs are ongoing. The combination of bezuclastinib, which targets activation-loop mutations, and sunitinib, which targets ATP–binding pocket mutations, is a rational approach aiming to target all resistance *KIT* mutations. This combination was deemed safe and active in a Phase 1b/2a study [42], and the results of the Phase III PEAK study of this combination versus sunitinib alone in the second-line are awaited [43]. A number of TKIs, such as IDRX-42 and NB003, are currently undergoing early-phase evaluation (Table 1).

Clinical trials with anti-PD1 and anti-CTLA4 inhibitors have shown unsatisfactory results in GIST so far. However, limited responses have been observed, which implies the need for the discovery of predictive biomarkers. Additional strategies, such as alternative immune checkpoint inhibitors, vaccines, and T-cell therapies, are also worthy of exploration [44].

Finally, there is progress in SDH-deficient wild-type GIST. The TKI olverembatinib has shown promising activity in a Phase I study conducted in China with 6 patients experiencing PR and 18 SD out of 26 patients with SDH-deficient GIST [45]. In addition, a preclinical study demonstrated that a patient-derived SDH-mutant GIST cell model was sensitive to the alkylating agent temozolomide (TMZ) due to the metabolic defects associated with deficiency of the SDH [46]. The SDH-mutant cells were also shown to have elevated levels of hypoxia-inducible factor 1 alpha (HIF1a) and hypoxia-inducible factor 2 alpha (HIF2α) with activation of the downstream targets [46]. In addition, the global DNA hyper-methylation associated with the SDH-deficient GIST has been shown to mediate insulator losses and topological reorganisation of the Fibroblast Growth Factor (*FGF)* and *KIT* loci which leads to induction of the *FGF4* and *FGF3* oncogenes and sensitivity to FGF receptor (FGFR) inhibition [47]. All this preclinical evidence led to the initiation of clinical trials with the HIF2a inhibitor belzutifan and TMZ alone or in combination with a DR5 agonist in SDH-deficient GIST. FGFR inhibition may also warrant clinical investigation. Ongoing clinical trials in SDH-deficient GIST are shown in Table 1.

## 3. Liposarcoma

### 3.1. Well-Differentiated and Dedifferentiated Liposarcoma

Liposarcoma (LPS) is one of the most common histologic subtypes of STS. Well-differentiated (WDLPS) and dedifferentiated liposarcoma (DDLPS) are considered to belong to the same disease spectrum and together represent the largest subgroup of liposarcomas [48]. Both WDLPS and DDLPS are associated with high-level amplification of the chromosomal 12q13-15 region, which contains the *MDM2*, *HMGA2*, and *CDK4* genes. *CDK4* is amplified less frequently than *MDM2* [49].

Chemotherapy has only moderate activity in WDLPS and DDLPS [50]; thus, the exploration of alternative therapeutic strategies is required. The amplification of *MDM2* and *CDK4* genes creates opportunities for therapeutic exploitation. MDM2 has an E3 ubiquitin ligase function and is a negative regulator of p53 by promoting its ubiquitination and proteasomal degradation and by directly blocking its N-terminal trans-activation domain [51]. The inhibition of the interaction of MDM2 with p53 is a rational therapeutic strategy that has been shown to lead to an increase in the levels of p53 and induce cell cycle arrest and apoptosis in cancer cells in vitro [52].

Milademetan, a small molecule inhibitor of MDM2, was tested in a Phase I trial in an intermittent dosing schedule of 3/14 days to mitigate haematologic toxicity. The most common drug-related all-grade adverse events were nausea (72.0%), thrombocytopenia (60.7%), fatigue (44.9%), and anaemia (35.5%). In the total population of patients with DDLPS, the disease control rate was 58.5%, and the median PFS was 7.2 (95% CI, 3.8–10.1) months across all dosing schedules [53]. However, the randomised Phase III MANTRA trial, which evaluated milademetan versus trabectedin in patients with unresectable or metastatic DDLPS with or without WDLPS component who had progressed on one or more prior systemic therapies, including at least one anthracycline-based therapy, failed to show superiority of milademetan. The median PFS was 3.7 months for milademetan versus 2.1 months for the standard of care trabectedin with a hazard ratio (HR): 0.80 (95% CI, 0.55–1.15; *p* = 0.22) [54].

Brigimadlin (BI907828), an oral MDM2 inhibitor, showed manageable toxicity and encouraging activity in patients with MDM2-amplified WDLPS and DDLPS. The most common treatment-related adverse events (TRAEs) were nausea (74.1%) and vomiting (51.9%), and the most common grade ≥ 3 TRAE were thrombocytopenia (25.9%) and neutropenia (24.1%) in keeping with the role of MDM2 in hematopoiesis. Of the 7 patients with WDLPS, 4 achieved a RECIST partial response, and 3 achieved stable disease, resulting in a 100% disease control rate, while 9 of the 12 patients with DDLPS achieved stable disease with a disease control rate of 75% [55]. The results of Brightline-1, a randomised open-label Phase II/III that evaluated whether brigimadlin is superior to doxorubicin in the first-line treatment of advanced/metastatic DDLPS are highly anticipated [56].

Additional MDM2 inhibitors have been evaluated in the past. Examples are AMG232 [57], SAR405838 [58], and MK8242 [59] in the context of early-phase clinical trials and RG7112 in an exploratory proof-of-mechanism study in patients with WDLPS and DDLPS [60]. These trials showed signals of efficacy, with stable disease in a subset of patients and, less frequently, partial responses. The main adverse events were due to gastrointestinal and haematologic toxicity.

Inhibition of CDK4 is another rational target in patients with WDLPS and DDLPS, given the amplification of the *CDK4* gene in approximately 90% of cases of WDLPS and DDLPS. The CDK4/6 inhibitor palbociclib in a dose and schedule of 125 mg daily for 21 days every 28 days resulted in a median PFS of 17.9 (95% CI, 11.9–24.0) weeks and PFS rate at 12 weeks of 57.2% (95% CI, 42.4–68.8%) in a Phase 2 trial in patients with advanced WDLPS and DDLPS [61]. The most common toxicity was neutropenia (grade 3: 33% and grade 4: 3%) without neutropenic fever [61]. Abemaciclib, another CDK4/6 inhibitor in a dose and schedule of 200 mg by mouth twice daily continuously, resulted in a median PFS of 30.4 (95% CI, 28.9-NE) weeks and PFS rate at 12 weeks of 76% (95% CI, 57–90%) in patients with advanced DDLPS in a Phase 2 study [62]. Grade 3–4 toxicity included anaemia (37%), neutropenia (20%), thrombocytopenia (17%), and diarrhoea (7%) [62]. These results were deemed promising and led to the initiation of a Phase 3 randomised double-blind study of abemaciclib versus placebo in patients with recurrent or metastatic DDLPS of any line of treatment. Patients who progress on placebo may cross over to abemaciclib [63] (Table 1).

The combination of CDK4/6 inhibitors with other targeted agents has been evaluated. Co-targeting MDM2 and CDK4 is a reasonable approach in WDLPS and DDLPS. The combination of the CDK4/6 inhibitor ribociclib with the MDM2 inhibitor siremadlin was assessed in patients with advanced WDLPS and DDLPS in a Phase Ib dose-escalation study [64]. In this trial, the regimen of a 3-week cycle of siremadlin once every 3 weeks and ribociclib 200 mg once daily (2 weeks on, 1 week off) was selected as the recommended dose of expansion. Dose-limiting toxicities (DLTs) occurred in 10 patients across all regimens, which were grade 3/4 haematologic events. One patient died due to haematologic toxicity. Of the 74 patients enrolled, 3 patients achieved a partial response, and 38 achieved stable disease [64].

Another investigational approach in WDLPS and DDLPS was the use of selinexor, a potent oral inhibitor of exportin 1 (XPO1), which facilitates the nuclear retention of tumour suppressor proteins. In liposarcoma cell lines, selinexor increased the expression of p53 without affecting MDM2 levels and increased the expression of p21, an inhibitor of CDK4 [65]. This made it an attractive agent in WDLPS and DDLPS, given the underlying amplification of *MDM2* and *CDK4* genes. However, it did not result in significant clinical benefit and has not gained regulatory approval. In particular, in the SEAL trial, patients with advanced DDLPS who had received two to five lines of treatment were randomised to selinexor or placebo in a 2:1 ratio. Treatment with selinexor led to a modest increase in median PFS (95% CI, 0.52 to 0.95; one-sided *p* = 0.011; median PFS 2.8 vs. 2.1 months respectively) with a hazard ratio (HR): 0.70. No significant improvement in overall survival was observed [66].

Immune checkpoint inhibitors have been tested in WDLPS and DDLPS. However, the regimens evaluated so far have shown limited efficacy. Pembrolizumab in the liposarcoma expansion cohort in the SARC028 trial resulted in an ORR of 10% and mPFS of 2 months (95% CI, 2–4) [30]. No response was observed in the 5 patients with WDLPS and DDLPS treated with either nivolumab or nivolumab plus ipilimumab in the Alliance trial [27]. More encouraging were the results of the combination of doxorubicin with pembrolizumab. In particular, in one Phase I/II study, 2 out of 4 patients with DDLPS achieved durable PR [67], and in another Phase II study, an ORR of 28.6% was documented in patients with liposarcoma [68].

Ongoing clinical trials with novel agents and combinations in WDLPS and DDLPS are shown in Table 1.

### 3.2. Myxoid Liposarcoma

Myxoid liposarcoma (MLPS) is another LPS subtype, comprising approximately 30% of the cases [69]. As opposed to WDLPS and DDLPS, it is characterised by the pathognomonic translocation t(12;16)(q13;p11) in almost 95% of cases, which results in the fusion oncogene *FUS::DDIT3*. The alternative fusion oncogene *EWSR1::DDIT3* is detected in approximately 5% of MLPS due to the translocation t(12;22)(q13;q12).

MLPS is more chemosensitive and radiosensitive than WDLPS and DDLPS [50,70]. MLPS also exhibits high sensitivity to trabectedin. In a preclinical study, trabectedin was shown to block the transactivating activity of the fusion oncoprotein *FUS-DDIT3* and derepress the adipocytic differentiation of the non-lipogenic tumour cells in MLPS xenografts [71]. Given preclinical evidence of potential synergism of trabectedin with radiotherapy, a Phase I trial was conducted, which showed that the combination of neoadjuvant trabectedin with radiotherapy (RT) (45 Gy) in 25 fractions is active and feasible in patients with localised MLPS without cumulative toxicity. The recommended dose for the Phase 2 study was determined to be 1.5 mg/m^2^ which is the same dose approved for trabectedin alone [72]. A follow-up Phase II study showed that 9 out of 41 patients (22%) achieved partial response, 5 out of 39 patients (13%) experienced complete pathologic response, and 20 out of 39 patients (51%) had 10% or less of viable remaining tumours. However, the primary endpoint of RECIST response in ≥70% of patients was not met, but the treatment was well tolerated [73]. An ongoing clinical trial that includes patients with MLPS is shown in Table 1.

MLPS demonstrates high expression of cancer testis antigens (CTA) such as the New York Esophageal Squamous Cell Carcinoma-1 (NY-ESO-1), preferentially expressed antigen in melanoma (PRAME), and melanoma-associated antigen-4 (MAGE-A4) [74,75,76]. This creates therapeutic opportunities for T Cell Receptor (TCR)-based therapies with engineered T cells and T-cell-activating bispecific antibodies, which engage the antigen-expressing tumour cells with the CD3 molecules on the T cells [77]. TCR-based therapies will be discussed in a separate section.

## 4. Synovial Sarcoma

Synovial sarcoma (SS) accounts for 5–10% of STS [4,78]. Genetically, it is marked by a pathognomonic translocation between chromosomes X and 18, t(X;18)(p11.2;q11.2) in >95% of cases [4,78]. This results in the SS18-SSX fusion oncoprotein, which alters gene expression by binding to the chromatin remodelling Switch/Sucrose Non-Fermentable (SWI/SNF) (BAF) complex. This leads to displacement of the wild-type SS18 and the tumour suppressor SWI/SNF (BAF)-related matrix-associated actin-dependent regulator of chromatin subfamily B member 1 (SMARCB1) [4,78]. The altered SWI/SNF (BAF) complex, in turn, promotes cellular proliferation [79].

Another hallmark of SS is the frequent and high expression of several cancer testis antigens (CTAs), such as MAGE-A4, NY-ESO-1, and PRAME, which may be mediated by the SS18-SSX oncoprotein [78,80].

SS is a chemosensitive tumour, but the overall prognosis in the advanced disease setting remains poor, which necessitates the development of new treatments [78].

Novel epigenetic treatment, such as targeting the bromodomain-containing protein 9 (BRD9), has been explored. Clustered, regularly interspaced short palindrome repeats (CRISPR)/Cas9 screening revealed a functional dependency of SS to BRD9 which is a component of the SS18-SSX containing BAF complexes critical for SS cell growth [81]. CFT8634, a bifunctional degradation activation compound (BiDAC™) of BRD9, was evaluated in a Phase 1/2 clinical trial (NCT05355753) for patients with locally advanced or metastatic SMARCB1-perturbed cancers, including SS and SMARCB1-null tumours. However, the program was halted due to a lack of sufficient efficacy. FHD-609, another potent and selective heterobifunctional protein degrader of BRD9, was evaluated in patients with advanced SS or advanced SMARCB1-loss tumours in a Phase I clinical trial (NCT04965753). However, this study is in partial clinical hold as of April 2023.

In addition, *BRAF* V600E mutation has been reported in rare cases of patients with SS. In particular, cases of two patients with intrathoracic monophasic SS positive for SS18-SSX2 were reported to harbour the *BRAF* V600E mutation [82]. One of the patients received treatment with the combination of BRAF/MEK inhibitors and dabrafenib/trametinib, which resulted in remission for 7.5 months [82]. This suggests that *BRAF* V600E is a potential therapeutic target for a small subset of patients with synovial sarcoma, and it warrants screening given the encouraging tumour-agnostic activity of the combination of dabrafenib with trametinib and its tumour-agnostic regulatory approval [83].

The expression of CTAs also makes SS a good candidate for TCR-based therapies [77], which will be discussed in the section below.

## 5. TCR-Based Therapies in MLPS and SS

TCR-based therapies are promising in MLPS and SS, given the high expression of CTAs in these tumours. These are autologous T cells expressing TCRs that target the epitopes of CTAs presented by the human leukocyte antigen (HLA) class I of the tumour cells [77]. CTAs have limited expression in normal tissue, and the most studied ones so far in STS are MAGE-A4 and NY-ESO-1.

Targeting MAGE-A4 led to the first FDA approval of TCR-based therapies in SS. In the open-label, Phase 2, SPEARHEAD-1 trial, patients with advanced SS and MLPS, positive for HLA-A*02 and MAGE-A4 tumour expression received afamitresgene autoleucel (afami-cel), an autologous TCR T-cell therapy after lymphodepleting chemotherapy containing fludarabine and cyclophosphamide. In the final results published in the Lancet in April 2024, the response rate was 37% (95% CI, 24–51) overall, 39% for patients with SS and 25% for patients with MLPS, while the median duration of response was 11.6 months (95% CI 4.4–18.0) in patients with SS and 4.2 months (2.9–5.5) in patients with MLPS [84]. The mPFS was 3.7 months (95% CI 2.8–5.6) overall, 3.8 months (2.8–6.4) in patients with SS, and 2.4 months (0.9–7.4) in patients with MLPS. These data confirm the efficacy of afami-cel in SS. Considering the small number of patients with MLPS (n = 8) compared to SS (n = 44), it is difficult to draw final conclusions for the MLPS population, which of note was associated with lower MAGE-A4 expression and higher disease burden [84]. On 2 August 2024, the FDA granted accelerated approval to afami-cel in adults with unresectable or metastatic synovial sarcoma who have received prior chemotherapy, are HLA-A*02:01P, -A*02:02P, -A*02:03P, or -A*02:06P positive, and whose tumor expresses the MAGE-A4 antigen.

Targeting NY-ESO-1 has shown encouraging activity as well. In the open-label Phase II trial IGNYTE-ESO, patients with advanced MLPS and synovial sarcoma (SS) who were HLA*02 positive with confirmed NY-ESO expression in ≥30% of tumour cells ≥ 2+ by IHC were treated with lete-cel, an engineered T-cell therapy targeting NY-ESO-1. Patients who were treatment naïve (substudy 1) or post-treatment with anthracycline chemotherapy (substudy 2) received lete-cel doses between 1–15 × 10^9^ of transduced T cells after receiving lymphodepleting chemotherapy [85]. With respect to safety, cytokine release syndrome was observed in 89% of patients, which was grade 1 and 2 in the majority of patients, without any case of grade 4 or 5. Immune effector cell-associated neurotoxicity syndrome (ICANS) was seen in less than 5% of patients and was grade 1 in all cases. Haematologic cytopenias were developed in 88% of patients and the majority were grade 3 or higher. In an interim analysis of efficacy, which included data from 45 patients with MLPS or SS who had at least 6 months follow-up, 18 out of 45 patients (40%) with MLPS or SS had RECISTv1.1 response by independent review with 2 CRs and 16 PRs. The response rate was 41% and 39% for patients with MLPS and SS, respectively. The primary analysis is expected to be performed in the first half of 2024.

Additional approaches to target NY-ESO-1 have been tested. In a Phase I/II trial, 8 Japanese patients with SS positive for HLA-A*02:01 or HLA-A*02:06 and NY-ESO-1 tumour expression received cyclophosphamide 750 mg/m^2^ on days −3 and −2 (induction period) followed by an infusion of a split dose of a cell suspension of 5 × 10^9^ (±30%) autologous T lymphocytes expressing NY-ESO-1 antigen-specific TCR gene and siRNA to inhibit the expression of endogenous TCR (product code: TBI-1301) on days 0 and 1. The ORR by central assessment was 50.0%, with best response PR for 4, SD for 1, and PD for 3 patients, respectively. Four out of eight patients developed cytokine release syndrome (CRS), which was grade 1 in one patient and grade 2 in three patients, respectively. No death was attributed to adverse events [86].

In another Phase I trial, autologous NY-ESO-1-specific TCR-T cells were combined with a subcutaneous vaccination of pullulan nanogel loaded with a synthetic long peptide antigen (LPA) containing NY-ESO-1_157-165_ with the intention of removing the need for lymphodepletion by boosting TCR-T cells [87]. A preclinical study by the same team showed that the pullulan nanogel/LPA vaccine led to an increase in the tumour infiltration of the infused TCR-T cells [87]. Three patients with refractory SS were treated. One patient experienced tumour shrinkage with durable response. One patient developed G1 CRS, and another one developed G2 CRS. No nonhematologic AEs of grade 3 or more were observed, and no death occurred during the trial assessment period [87]. 

## 6. Leiomyosarcoma

Leiomyosarcoma (LMS) is one of the most frequent STS subtypes, representing 10–15% of all cases. It is characterised by significant genetic heterogeneity [88]. LMS lacks a single defining targetable genetic alteration and is associated with extensive DNA copy number changes, including chromothripsis and whole genome duplications [6]. Common genetic alterations include mutations or deletions in the tumour suppressors RB1, TP53, and PTEN and alterations in telomere maintenance genes such as ATRX. In addition, alterations in genes involved in DNA repair mechanisms are frequently encountered, in particular in uterine LMS [6,88].

Current treatment with either single-agent chemotherapy or pazopanib has moderate activity [89]. Recently, combination chemotherapy with doxorubicin and trabectedin followed by trabectedin maintenance was shown to have superior efficacy compared to doxorubicin alone in a Phase 3 LMS04 trial with a mOS of 33 versus 24 months (HR: 0.65; 95% CI, 0.44–0.95) and mPFS 12 versus 6 months (HR: 0.37; 95% CI, 0.26–0.53) but at the expense of increased toxicity [90]. An alternative approach under investigation is the combination of doxorubicin with lurbinectedin, another marine-derived drug, a synthetic analogue to trabectedin, followed by lurbinectedin maintenance. This combination is compared with doxorubicin alone in the Phase IIb/III SaLuDo study (NCT06088290) which is currently recruiting (Table 1).

Novel strategies beyond chemotherapy are currently being explored. Given the defects in the homologous recombination repair pathway in a subset of patients with LMS, treatment with the poly (ADP-ribose) polymerase (PARP) inhibitor olaparib is a rational approach. The combination of olaparib with temozolomide was assessed in patients with uterine LMS in a Phase 2 study. The overall ORR was 27% (6 of 22), and the mPFS was 6.9 months (95% CI, 5.4-NE). Haematologic toxicity was common with grade 3/4 neutropenia and thrombocytopenia in 75% and 32% of patients, respectively, but it was manageable with dose reduction. Of note, an exploratory analysis showed that patients with homologous recombination deficient (HRD) tumours, as determined by a RAD51 foci formation assay, had prolonged mPFS as compared to patients with proficient tumours (11.2 vs. 5.4 months) [91]. A randomised Phase 2/3 Study of the combination of olaparib with temozolomide versus the investigator’s choice for patients with advanced uterine LMS after progression on prior chemotherapy is currently recruiting (Table 1). Inhibition of the ataxia telangiectasia and Rad3-related (ATR) may be another putative strategy for LMS with HRD, given its fundamental role in the DNA damage response [92]. Targeting ATR in Soft-tissue Sarcomas (TARSARC) is an ongoing Phase 2 randomised non-comparative trial of gemcitabine versus gemcitabine combined with the ATR inhibitor berzosertib in patients with advanced or metastatic leiomyosarcoma [93] (Table 1).

Treatment with immune checkpoint inhibitors alone has limited activity. No objective response was reported with the anti-PD1 pembrolizumab in 10 patients with LMS in the Phase 2 SARC028 trial [28]. One objective response was detected with the anti-PD1 nivolumab in 15 LMS patients, and 2 objective responses were reported with nivolumab combined with the anti-CTLA4 ipilimumab in 14 LMS patients in the Phase 2 Alliance (A091401) trial [27].

However, certain clinical trials have recently shown promising results with the combination of immune checkpoint inhibitors (ICI) with chemotherapy. The combination of ipilimumab, nivolumab, and trabectedin was assessed in a Phase 1/2 trial (SAINT) for previously untreated patients with advanced STS. A total of 26 of the 101 enrolled patients had LMS, and 22 of them were evaluable for efficacy. The ORR was 31.6%, the DCR was 89.5%, the mPFS was 7.4 (1.2–33.6) months, the mOS was 36.1 (1.6–45.8) months, the 6-month PFS rate was 63.2%, and the 6-month OS rate was 89.5% [94]. In the Phase 2 study by Livingston et al., the combination of pembrolizumab with doxorubicin resulted in 40% PR and 60% SD as the best response in the LMS cohort of 10 patients. In the total STS population of 30 patients, the mPFS was 5.7 (95% CI, 4.1–8.9) months, and the mOS was 17.0 (95% CI, 9.9–NE) months [68]. In a Phase 1b trial, the combination of doxorubicin and dacarbazine plus nivolumab resulted in PR in 9 patients (56.2%), SD in 6 patients (37.5%), and PD in 1 patient (6.3%), while the mPFS was 8.67 (95% CI: 7.96–9.37) months in the 16 efficacy-evaluable patients [95]. In the Phase 1 GEMMK trial, the combination of pembrolizumab with gemcitabine resulted in SD in 8 out of 11 patients with LMS as the best response [96]. However, the combination of eribulin with pembrolizumab resulted in an ORR of only 5.3% in the 19 patients in the LMS cohort, with 1 patient having experienced PR and 5 SD. The PFS at 12 weeks was 42.1% (90% CI: 27.0–65.5%), and the study did not meet its primary endpoint of 60% PFS at 12 weeks [97]. Results of clinical trials with ICI in STS are shown in Table 2.

## 7. Undifferentiated Pleomorphic Sarcoma (UPS)

Undifferentiated pleomorphic sarcoma (UPS), formerly known as malignant fibrous histiocytoma (MFH), is another frequent STS subtype, accounting for approximately 15% of cases. It is a diagnosis of exclusion when other lines of differentiation have been ruled out with immunohistochemistry (IHC) and molecular techniques. UPS is genomically complex and is often characterised by alterations of the *TP53*, *RB1*, cyclin-dependent kinase inhibitor 2A (*CDKN2A*), *PTEN,* and *ATRX* genes [110]. A high throughput profiling with IHC, RNA-sequencing, whole exome-sequencing, mass spectrometry, and radiomics identified two main groups of UPS, one enriched in genes involved in stemness and development, including *FGFR2,* and a second group enriched in genes involved in immunity [111]. Tumours belonging to the second group were characterised by significantly higher levels of CD8+ infiltrating lymphocytes as determined by IHC (immune-high), while cell lines and xenograft models derived from the first group (immune-low) exhibited sensitivity to FGFR inhibition [111].

The findings of high immune infiltration may explain the response to ICI in a subset of UPS. Based on the final results of the expansion cohorts of the SARC028 Phase 2 trial, pembrolizumab led to an ORR of 23%, mPFS of 3 months (95% CI: 2–5), mOS of 12 months (95% CI: 7–34), and 12-week PFS rate of 50% (95% CI: 35–65) in the UPS cohort [30]. These results deserve further validation in a randomised trial. However, in the Alliance trial (A091401), the response rate was only 8% and 14%, and the mPFS 1.5 (1.4-NE) months and 2.7 (1.5-NE) months with nivolumab and combination of nivolumab/ipilimumab, respectively [112].

There are some encouraging results of the combination of ICI with chemotherapy in UPS. In the Phase 2 trial by Livingstone et al., the combination of pembrolizumab with doxorubicin resulted in PR in all 4 patients with UPS (ORR: 100%) [68] and in the Phase 1/2 trial by Pollack et al.l, 2 out of the total 3 patients with UPS had a durable PR with the combination of pembrolizumab with doxorubicin [67]. In the Phase 1 GEMMK study, all 2 patients with UPS experienced PR [96]. In the Phase 2 study of eribulin plus pembrolizumab, the mPFS was 12.6 weeks, and the 12-week PFS rate was 62.5% in the 8 patients with UPS [103]. The Phase 1/2 SAINT study showed that the best response was CR in one, surgical CR in another one, SD in 5, and PD in 2 patients with UPS, respectively, with the combination of ipilimumab, nivolumab, and trabectedin in the first-line [102].

An indicative ongoing clinical trial in UPS is the ENVASARC study of the anti-PDL1 envafolimab and combination of envafolimab with ipilimumab (Table 1).

## 8. Recent Advances in Other Rare STS Subtypes

### 8.1. Angiosarcoma

Angiosarcoma is a rare and aggressive tumour accounting for approximately 1% of all STS [113]. It is relatively chemosensitive, but the overall prognosis is poor. The Angiosarcoma Project shed light on the molecular pathogenesis of angiosarcoma and revealed recurrent genetic alterations with therapeutic potential by analysis of 47 samples from 36 patients [114]. Some of the most frequent mutations were in the *TP53* and *KDR* genes, which were mutually exclusive, with 8 out of 9 *KDR* mutations being detected in primary breast angiosarcoma samples. *PIK3CA* was another frequently altered gene. Nine out of ten of these mutations occurred in primary breast angiosarcoma and suggested sensitivity to PI3Ka inhibition [114]. It was also demonstrated that angiosarcoma of the head, neck, face, and scalp (HNFS) was associated with significantly higher tumour mutational burden (TMB) compared to angiosarcoma from other sites. In addition, all nine HNFS samples with high TMB were characterised by a mutational signature associated with damage from ultraviolet (UV) light [114]. Notably, 3 out of 10 HNFS patients received off-label treatment with ICI against programmed cell death 1 (PD-1) protein, and 2 of them had a durable response. On the contrary, none of the 3 patients with non-HNFS angiosarcoma who received ICI treatment gained clinical benefit [114]. Additional clinical evidence supports the efficacy of ICI treatment in this subset of patients. A retrospective analysis of 7 patients with angiosarcoma who received ICI therapy in a single institution in the context of a clinical trial or off-label showed that 5 patients had an objective response, of which 4 had HNFS angiosarcoma [32]. In addition, the combination of ipilimumab and nivolumab in the angiosarcoma cohort in the SWOG S1609 study showed that 3 out of 5 patients with angiosarcoma of the scalp or face had an objective response, and the ORR in all 16 evaluable angiosarcoma patients was 25%. Indicative of ongoing clinical trials are a Phase 2 study with cemiplimab in patients with angiosarcoma and a Phase 1 study of AGEN1181, an Fc-engineered anti-CTLA-4 monoclonal antibody as monotherapy and in combination with AGEN2034 (Balstilimab), an anti-PD-1 monoclonal antibody, in subjects with advanced cancer, including angiosarcoma. Additional all-comer STS clinical trials with ICI, which include patients with angiosarcoma, are ongoing (Table 1).

### 8.2. Epithelioid Sarcoma

Epithelioid sarcoma (ES) is an ultra-rare tumour representing less than 1% of STS characterised by loss of the tumour suppressor INI1 (SMARCB1), a subunit of the SWI/SNF chromatin complex, on IHC in 90% of cases [115]. INI1 loss drives oncogenesis and leads to dependence on the transcriptional repressor enhancer of zeste homolog 2 (EZH2), which creates a therapeutic opportunity. Tazemetostat, an EZH2 inhibitor, was tested in patients with locally advanced or metastatic epithelioid sarcoma with documented loss of INI1 expression with IHC or biallelic *SMARCB1* alterations in a Phase 2 trial. Tazemetostat resulted in ORR of 15%, mPFS of 5.5 (95% CI, 3.4–5.9) months and mOS of 19.0 (11.0–NE) months. These results are comparable to those achieved with chemotherapy, but tazemetostat is better tolerated. Based on these data, the FDA granted approval for tazemetostat for adults and paediatric patients aged 16 years and older with metastatic or locally advanced epithelioid sarcoma not eligible for complete resection. In addition, the results of the randomised Phase 3 trial of doxorubicin plus tazemetostat versus doxorubicin alone as front-line therapy for advanced epithelioid sarcoma are awaited [116]. Another area worthy of exploration is the use of ICI. We and others have reported cases of patients with ES treated with ICI either off-label or in the context of a clinical trial with signs of activity [117,118,119]. In addition, pre-clinical and clinical evidence suggests that a subset of *SMARCB1*-deficient tumours may be immunogenic [120]. Clinical trials of tazemetostat in combination with nivolumab and ipilimumab in children with INI1-negative tumours, nivolumab and ipilimumab in children and young adults with INI1-negative tumours, and a combination of atezolizumab with the anti-TIGIT tiragolumab in INI1-deficient tumours, are currently ongoing and include patients with ES (Table 1).

### 8.3. Alveolar Soft Part Sarcoma (ASPS)

Alveolar soft part sarcoma (ASPS) is an ultra-rare STS subtype representing less than 1% of all STS [121]. It is characterised by the *ASPSCR1::TFE3* fusion gene due to the t(x;17) (p11,q25) translocation, which has been shown to promote tumour growth and angiogenesis [122]. Chemotherapy has limited efficacy, but treatment with antiangiogenic TKIs and immunotherapy have demonstrated activity [121]. Sunitinib, pazopanib, cabozantinib, cediranib, and anlotinib are examples of active TKIs in ASPS [121,123,124,125,126,127]. Recent studies highlighted the activity of ICI in ASPS. In a Phase 2 trial of atezolizumab in advanced ASPS, an objective response was observed in 19 of the 52 evaluable patients with one CR and ORR of 37% (95% CI, 24–51). The best response in the other 33 patients was one PR, which was not confirmed, one PR according to iRECIST criteria, SD in 28 patients, and PD in 3 patients. The mPFS was 20.8 months, the median duration of response (mDOR) was 24.7 months, and the safety profile was consistent with what has been previously reported with atezolizumab monotherapy [29]. Based on these results, the FDA approved atezolizumab for adults and paediatric patients 2 years of age and older with unresectable or metastatic ASPS. In the Phase 2 AcSé Pembrolizumab trial, a basket study of pembrolizumab in patients with rare and ultra-rare sarcomas, the best response was a CR in 1 patient, PR in 7 patients, SD in 3 patients, PD in 3 patients, and the mPFS was 6.6 (5.5-NE) months in the 14 patients with ASPS [119]. The combination of axitinib with pembrolizumab was also promising in the ASPS population in a single-centre Phase 2 trial for patients with advanced or metastatic sarcomas. In the 11 evaluable patients with ASPS, 6 patients achieved PR, resulting in an ORR of 54.5% (95% CI, 24.6–81.9), 2 patients achieved SD, and the mPFS was 12.4 (95% CI 2.7–22.3) months [105]. However, these results require evaluation in a randomised clinical trial to assess whether the combination of a TKI with ICI outperforms monotherapy. The combination of durvalumab (anti-PD-L1) and tremelimumab (anti-CTLA-4 drug) was also tested in ASPS in a single-centre Phase 2 trial of patients with advanced or metastatic sarcomas. The ORR by irRECIST in the 10 patients with ASPS was 40% (95% CI, 12–74), with 2 patients having achieved CR. Pseudoprogression was also noted, which was more prominent in ASPS and indicates the need for longer treatment duration and confirmatory scans [98]. Ongoing clinical trials in ASPS include trials of atezolizumab alone or plus selinexor, atezolizumab alone or plus bevacizumab, and combination of sunitinib and nivolumab in a multicohort study which includes ASPS and combination of AB122 (anti-PD1) with TAS-115 (TKI) in a Phase 1 platform trial with an ASPS cohort (Table 1).

### 8.4. NTRK Fusion Sarcomas

Neurotrophic tyrosine receptor kinase (*NTRK*) fusion genes represent rare tumour-agnostic oncogenic drivers detected in more than 90% of infantile fibrosarcoma cases but in less than 1% of all STS [128]. The *NTRK*-rearranged spindle cell neoplasms, excluding infantile fibrosarcoma, which have distinct clinicopathologic features, are an “emerging entity” based on the WHO 2020 classification of soft tissue tumours in consideration of the advancement in NTRK inhibition [1,129]. The NTRK inhibitors larotrectinib and entrectinib are well-tolerated and were shown to provide high objective response rates with rapid and durable responses in a tumour-agnostic fashion [23,24,130]. Both drugs have gained FDA approval. Second-generation NTRK inhibitors are in development for patients who develop resistance mutations to current inhibitors [131].

### 8.5. PEComa

Perivascular Epithelioid Cell Tumour (PEComa) encompasses a group of rare benign and malignant mesenchymal neoplasms [132,133]. Malignant PEComa is extremely rare and is frequently associated with germline or somatic loss-of-function mutations in the tuberous sclerosis 1 (*TSC1*) or tuberous sclerosis 2 (*TSC2*) genes, which leads to activation of the mTOR pathway, suggesting sensitivity to mTOR inhibitors. The activity of the mTOR inhibitor nab-sirolimus in malignant PEComa was demonstrated in the AMPECT Phase 2 study, which showed an ORR of 38.7% (95% CI, 21.8 to 57.8), mPFS of 10.6 (95% CI, 5.5 to 41.2) months, and mDOR of 39.7 (95% CI, 6.5-NE) months [134,135]. Mucositis, fatigue, and rash were the most common side effects. Correlative studies showed that the presence of inactivating TSC2 mutations and phosphorylation of S6 ribosomal protein were predictors of response to nab-sirolimus [134,135]. The FDA approved nab-sirolimus for adults with locally advanced unresectable or metastatic PEComa.

## 9. Other Soft Tissue Tumours

### Desmoid Tumours

Desmoid tumours (DT) or aggressive fibromatosis are locally aggressive tumours without metastatic potential [136]. The majority of cases are sporadic and develop due to mutations in the Catenin Beta 1 (CTNNB1) gene, while the rest (approximately 10%) are associated with the familial adenomatous polyposis (FAP) syndrome caused by germline inactivating mutations in the adenomatous polyposis coli (APC) gene involved in the downregulation of β-catenin. There is a cross-talk between the Wnt/β-catenin and Notch signalling pathway; thus, suppression of Notch signalling with γ-secretase inhibitors is a rational approach. In the Phase 3, double-blind, randomised, placebo-controlled trial DeFi, the γ-secretase inhibitor nirogacestat was shown to improve PFS (HR: 0.29, 95% CI, 0.15–0.55; *p* < 0.001) and ORR (41% versus 8%; *p* < 0.001) over placebo [137]. There was also improvement in patient-reported outcomes, including pain, physical or role functioning, and health-related quality of life [137]. The most frequent adverse events were diarrhoea, nausea, fatigue, hypophosphatemia, and maculopapular rash [137]. Ovarian dysfunction was observed in 75% of women of childbearing potential receiving nirogacestat but resolved in 74% of them [137]. The FDA approved nirogacestat for adult patients with progressing desmoid tumours who require systemic treatment. RINGSIDE is another Phase 2/3 randomised trial with the γ-secretase inhibitor AL102 that has completed recruitment (Table 1).

## 10. Conclusions/Perspective

The rarity, along with the histological and biological heterogeneity of STS, makes the development of new therapeutics challenging. One of the main challenges is that most STS subtypes, with the exception of GIST, have either complex karyotypes without a single genetic driver mutation or non-targetable genetic alterations [138]. The way to foster the development of novel therapeutics in STS could lie in the axes below.

First, it is critical to identify new therapeutic targets. Genome-wide CRISPR/Cas9 screens can help to prioritise drug targets by identifying vulnerabilities in sarcoma [139]. This strategy can be useful in loss-of-function genetic alterations in tumour suppressor genes that are not directly targetable by identifying synthetic lethality gene pairs. Consortiums such as the Cancer Dependency Map (DepMap) can help in this direction [140]. However, it is essential to establish clinically relevant models which reflect the molecular landscape of patient tumours [141,142,143].

Along with the discovery of novel therapeutic targets, there is a requirement to identify predictive biomarkers. An example is the selection of patients with STS who are likely to respond to ICI treatment. Some promising findings in this area came from a large analysis of the gene expression profile in 608 STS tumours. This revealed five distinct phenotypes, of which two were classified as ‘’immune-high’’. In an independent validation cohort of 93 STS tumours, one of these two immune-high groups was characterised by the presence of tertiary lymphoid structures (TLS) enriched by T cells, follicular dendritic cells, and, in particular, B cells. When the pathology specimens of the immunotherapy trial in sarcoma SARC028 were analysed, it was shown that those belonging to this particular class were associated with a favourable response to the ICI pembrolizumab [144]. These findings led to an amendment of the PEMBROSARC Phase 2 trial, which is a combination of pembrolizumab with low-dose cyclophosphamide, and a new cohort of TLS-positive STS patients was included. In this cohort, the ORR was 30% (95% CI, 14.7–49.4), and the 6-month non-progression rate (NPR) was 40% (95% CI, 22.7–59.4), whereas the previously reported ORR and NPR in all-comer cohorts were only 2.4% (95% CI, 0.1–12.9) and 4.9% (95% CI, 0.6–16.5), respectively [145]. These results suggest that TLS could serve as a potential predictive biomarker for the selection of sarcoma patients who may benefit from ICI therapy.

Another avenue that requires systematic efforts is targeting difficult to ‘drug’ targets. Approximately 20% of STS are driven by oncogenic gene fusions. The vast majority of them do not involve targetable kinases but transcription factors, which lack defined pockets for binding of conventional ligands [138,146]. An area that holds promise is the regulation of disease-related proteins with direct protein degradation by using proteolysis-targeting chimaeras (PROTAC) and molecular glue degraders (MGDs), which employ the cellular ubiquitin-proteasome system for targeted degradation [146]. This technology can have applications in translocation-associated sarcoma and there are notable scientific efforts towards this direction. The multi-institutional research team KOODAC, which includes sites in Austria, France, Germany, the UK, and the US, has the challenge to develop PROTACs and MGDs to target the oncoproteins that drive paediatric tumours, including Ewing sarcoma (mediated by the EWSR1-FLI1 fusion) and fusion-positive rhabdomyosarcoma (PAX3-FOXO1 or PAX7-FOXO1) [147].

Finally, there is a need for a change in perspective from histology- to biology-driven treatment in sarcoma clinical trials. Tumour-agnostic therapeutics have paved the way, and methodological expertise on novel clinical trial designs is available [148]. A valuable source of knowledge in rare malignancies when clinical trials are not feasible is the growing real-world evidence of patients treated with off-label medications when there is a biologic rationale [149]. The shift in mindset toward biology-directed treatments and the importance of the partnership of academia with industry cannot be overstated.

## Figures and Tables

**Table 1:** A non-exhaustive list of representative active clinical trials in soft tissue sarcomas.

Clinical Trial Identifier	Title	Phase	Biologic Rationale	Population	Estimated Completion Date
GIST					
NCT05489237	A First-in-human (FIH) Study of IDRX-42 in Participants With Metastatic and/or Unresectable Gastrointestinal Stromal Tumours (GIST)	1/1b	Novel TKI	GIST	September 2026
NCT04936178	A Multicenter Phase 1, Open-Label Study of NB003 to Assess Safety, Tolerability, Pharmacokinetics, and Efficacy in Patients With Advanced Malignancies	1	Novel TKI	GIST	December 2025
NCT05208047	(Peak) A Phase 3 Randomised Trial of CGT9486 + Sunitinib vs. Sunitinib in Subjects With Gastrointestinal Stromal Tumours	3	Combination of two TKIs for a broader control of resistance mutations	GIST	September 2026
NCT04924075	Belzutifan/MK-6482 for the Treatment of Advanced Pheochromocytoma/Paraganglioma (PPGL), Pancreatic Neuroendocrine Tumour (pNET), Von Hippel-Lindau (VHL) Disease-Associated Tumours, Advanced Gastrointestinal Stromal Tumour (wt GIST), or Solid Tumours With HIF-2α Related Genetic Alterations (MK-6482-015)	2	To target the elevated HIF2a expression in SDH-deficient GIST	Wild-type GIST PPGL pNET (VHL) disease-associated tumors Tumours with HIF-2α related genetic alterations	February 2027
NCT05661643	The Efficacy and Safety of Temozolomide in SDH-deficient GIST (GIST)	2	To target the selectively sensitive to temozolomide SDH-deficient GIST	Wild-type GIST	December 2027
NCT03715933	Phase 1 Study of INBRX-109 in Subjects With Locally Advanced or Metastatic Solid Tumours Including Sarcomas	1	SDH-deficient solid tumours or GIST: to target the selectively sensitive to temozolomide SDH-deficient GIST in combination with a human death receptor 5 (DR5) agonist, given that temozolomide increases DR5 expression	GIST SDH-deficient tumours Sarcoma Pleural mesothelioma Gastric adenocarcinoma CRC Pancreatic adenocarcinoma	July 2026
DDLPS					
NCT04967521	SARC041: Study of Abemaciclib Versus Placebo in Patients With Advanced Dedifferentiated Liposarcoma	3	To target CDK4	DDLPS	November 2024
NCT05827614	Study of the CHK1 Inhibitor BBI-355, an ecDNA-directed Therapy (ecDTx), in Subjects With Tumours With Oncogene Amplifications (POTENTIATE)	1/2	To exploit the replication stress-associated with oncogene amplifications in extrachromosomal DNA	Tumours with evidence of oncogene amplification, including liposarcoma	September 2027
NCT05694871	Testing the Addition of Cemiplimab to Palbociclib for the Treatment of Advanced Dedifferentiated Liposarcoma	2	To combine inhibition of CDK4 with anti-PD-1	DDLPS	May 2027
LMS					
NCT05432791	Testing Olaparib and Temozolomide Versus the Usual Treatment for Uterine Leiomyosarcoma After Chemotherapy Has Stopped Working	2/3	To combine parp inhibition with temozolomide and target homologous recombination deficiency	Uterine LMS	March 2030
NCT04807816	Targeting ATR in Soft-tissue Sarcomas (TARSARC)	2	To leverage DNA damage response by combining ATR inhibition with chemotherapy	LMS	April 2026
NCT06088290	Study of Lurbinectedin in Combination With Doxorubicin Versus Doxorubicin Alone as First-line Treatment in Participants With Metastatic Leiomyosarcoma (SaLuDo)	2b/3	Combination of 2 chemotherapeutics followed by maintenance chemotherapy	LMS	November 2026
UPS & MFS					
NCT04480502	ENVASARC: Envafolimab And Envafolimab With Ipilimumab In Patients With Undifferentiated Pleomorphic Sarcoma Or Myxofibrosarcoma (ENVASARC)	2	To combine anti-PD-L1 with anti-CTLA-4	UPS MFS	June 2024
NCT03425279	A Phase 1/2 Dose Escalation and Dose Expansion Study of Mecbotamab Vedotin (BA3011) Alone and in Combination With Nivolumab in Adult and Adolescent Patients 12 Years and Older With Advanced Solid Tumours	1/2	To selectively target tumour cells with anti-AXL antibody-drug conjugate alone and in combination with anti-PD-1	UPS MFS	December 2024
Angiosarcoma					
NCT03860272	A Phase 1 Study of AGEN1181, an Fc-Engineered Anti-CTLA-4 Monoclonal Antibody as Monotherapy and in Combination With AGEN2034 (Balstilimab), an Anti-PD-1 Monoclonal Antibody, in Subjects With Advanced Cancer	1	Combination of anti-PD-1 with Fc-engineered anti-CTLA-4 to harness a novel mechanism for enhanced FcγR-dependent functionality	Angiosarcoma HCC NSCLC Prostate cancer Breast cancer	December 2026
NCT05799612	Phase I Study of TH1 Dendritic Cell Immunotherapy for the Treatment of Cutaneous Angiosarcoma	1	To combine paclitaxel, mRNA plus lysate-loaded dendritic cell vaccine, pegylated-interferon alpha, and filgrastim	Cutaneous head and neck angiosarcoma	December 2029
ES and INI1-deficient tumours					
NCT04416568	Study of Nivolumab and Ipilimumab in Children and Young Adults With INI1-Negative Cancers	2	To combine anti-PD-1 and anti-CTLA-4 in INI1-negative or SMARCA4-deficient tumours	ES Other INI1- or SMARCA4-deficient malignant tumours Chordoma (poorly differentiated or dedifferentiated) ATRT MRT RTK	October 2025
NCT05407441	TAZNI: A Phase I/II Combination Trial of Tazemetostat With Nivolumab and Ipilimumab for Children With INI1-Negative or SMARCA4-Deficient Tumours	1/2	To combine EZH2 inhibition with anti-PD-1 and anti-CTLA-4 in INI1-negative or SMARCA4-deficient tumours	ES Other INI1- or SMARCA4-deficient malignant tumours Chordoma (poorly differentiated or dedifferentiated) ATRT MRT RTK	February 2029
NCT05286801	Tiragolumab and Atezolizumab for the Treatment of Relapsed or Refractory SMARCB1 or SMARCA4 Deficient Tumours	1/2	To combine anti-TIGIT and anti-PD-L1 immune checkpoint inhibitors	ES Other SMARCB1- or SMARCA4-deficient malignant tumours Chordoma (poorly differentiated) ATRT MRT RMC	September 2025
ASPS					
NCT03141684	Testing Atezolizumab Alone or Atezolizumab Plus Bevacizumab in People With Advanced Alveolar Soft Part Sarcoma	2	To combine immunotherapy (anti-PD-L1) with antiangiogenic (anti-VEGFA)	ASPS	October 2025
NCT05333458	Testing Atezolizumab With or Without Selinexor in Patients ≥ 18 Years Old With Alveolar Soft Part Sarcoma, the Axiom StudyA Phase II study, with a safety lead-in, to evaluate ATX-101, a peptide drug targeting	2	To combine immunotherapy (anti-PD-L1) with selective inhibitors of nuclear export (selinexor)	ASPS	May 2025
NCT04999761	Platform Study of AB122-Based Treatments in Patients With Advanced Solid Tumours	1	ASPS cohort: to combine anti-PD1 (AB122) immunotherapy with an antiangiogenic TKI (TAS-115)	ASPS Pancreatic cancer CRC NSCLC Gastric cancer	May 2026
Miscellaneous sarcomas					
NCT03277924	Trial of Sunitinib and/or Nivolumab Plus Chemotherapy in Advanced Soft Tissue and Bone Sarcomas (ImmunoSarc)	1/2	Cohort 1–6: To combine an antiangiogenic TKI (sunitinib) with anti-PD-1 (nivolumab) Cohort 7: To combine chemotherapy with anti-PD-1 Cohort 8: To combine chemotherapy with anti-PD-1	Cohort 1–6: DDCS, EMC, vascular sarcoma, SFT, ASPS, CCS Cohort 7: UPS, LMS Cohort 8: osteosarcoma	June 2025
NCT05182164	Combination of Pembrolizumab and Cabozantinib in Patients With Advanced Sarcomas (PEMBROCABOSARC)	2	To combine a broad antiangiogenic TKI with anti-PD-1	UPS Osteosarcoma Ewing sarcoma	October 2025
NCT04668300	A Phase II Multi-Arm Study to Test the Efficacy of Oleclumab and Durvalumab in Multiple Sarcoma Subtypes	2	To combine immunotherapy with anti-PD-L1 and anti-CD73	Angiosarcoma DDLPS Osteosarcoma	June 2024
NCT05492682	A Study to Evaluate the Safety and Immune Activity of PeptiCRAd-1 in Combination With Pembrolizumab in Patients With Injectable Solid Tumours in Indications Known to Express NY-ESO-1 and MAGE-A3	1	Oncolytic virus coated with MAGE-A3 and NY-ESO-1 peptides to direct cytotoxic T cells to target tumours expressing these antigens	SS MLS Melanoma TNBC NSCLC CRC	September 2025
Desmoid tumours					
NCT04871282	A Study of AL102 in Patients With Progressing Desmoid Tumours (RINGSIDE)	2/3	To block notch signalling with a γ-secretase inhibitor (AL102)	Desmoid tumours	February 2025

TKI: tyrosine kinase inhibitor, GIST: gastrointestinal stromal tumour, DDLPS: dedifferentiated liposarcoma, SS: synovial sarcoma, UPS: undifferentiated pleomorphic sarcoma, MFS: myxofibrosarcoma, MLS: myxoid liposarcoma, LMS: leiomyosarcoma, SFT: solitary fibrous tumour, ASPS: alveolar soft part sarcoma, CCS: clear cell sarcoma, DDCS: dedifferentiated chondrosarcoma, EMC: extraskeletal myxoid chondrosarcoma, TNBC: triple negative breast cancer, NSCLC: non-small cell lung cancer, colorectal cancer: CRC, PPGL: pheochromocytoma/paraganglioma, pNET: pancreatic neuroendocrine tumour, VHL: Von Hippel-Lindau, HCC: hepatocellular cancer, ES: epithelioid sarcoma, ATRT: atypical teratoid rhabdoid tumour, MRT: malignant rhabdoid tumour, RTK: rhabdoid tumour of the kidney, RMC: renal medullary carcinoma.

**Table 2:** A list of completed clinical trials with immune checkpoint inhibitors in sarcomas.

Title/Regimen	Phase	Population	Outcome	Reference
Pembrolizumab in advanced soft-tissue sarcoma and bone sarcoma (SARC028): a multicentre, two-cohort, single-arm, open-label, Phase 2 trial	2	UPS, DDLPS, SS, LMS	ORR: 18% (95% CI, 7–33%), mPFS: 18 weeks (95% CI, 8–21), 12-week PFS rate: 55% (95% CI, 40–70)	[28]
A non-comparative multi-center randomized Phase II study of nivolumab +/− ipilimumab for patients with metastatic sarcoma (Alliance A091401)	2	Multiple sarcoma histologies	Monotherapy armORR: 5% (92% CI, 1–15%), mPFS:1.7 months (95% CI, 1.4–4.3) Combination arm ORR: 16% (92% CI, 7–29%),mPFS: 4.1 months (95% CI, 2.6–4.7)	[27]
Durvalumab plus tremelimumab in advanced or metastatic soft tissue and bone sarcomas: a single-centre Phase 2 trial	2	LPS, LMS, angiosarcoma, UPS, synovial sarcoma, osteosarcoma, alveolar soft-part sarcoma, chordoma, and other sarcomas	ORR: 12% (95% CI, 5–24), mPFS: 2.8 months (95% CI, 1.8–6.4), 12-month PFS: 28% (95% CI, 17–40)	[98]
Trabectedin plus Durvalumab in Patients with Advanced Pretreated Soft Tissue Sarcoma and Ovarian Carcinoma (TRAMUNE): An Open-Label, Multicenter Phase Ib Study	1b	LMS, DDLPS, other	ORR: 7% (95% CI, 0.2–33.9), mPFS: 2 months, 1-year PFS rate: 14.3% (95% CI, 2.3–36.6)	[99]
A Phase 1/2 Trial Combining Avelumab and Trabectedin for Advanced Liposarcoma and Leiomyosarcoma	1/2	LMS, LPS	ORR: 13%, 6-month PFS rate: 52% (95% CI, 35–77), mPFS: 8.3 months (95% CI, 2.5–infinity)	[100]
Efficacy and safety of nivolumab and trabectedin in pretreated patients with advanced soft tissue sarcomas (STS): Results of a Phase II trial of the German Interdisciplinary Sarcoma Group (GISG-15, NitraSarc)	2	Group A: LMS, LPS Group B: pleomorphic, spindle cell, fibromyxoid, synovial, epithelial	Group A: 6-month PFS: 47.6%, mPFS: 5.5 months Group B: 6-month PFS: 14.6%, mPFS: 2.3 months	[101]
SAINT: A Phase I/Expanded Phase II Study Using Safe Amounts of Ipilimumab, Nivolumab and Trabectedin as First-Line Treatment of Advanced Soft Tissue Sarcoma	1/2	LMS, LPS, UPS, rhabdomyosarcoma, SS, other	ORR: 25.3%, mPFS: 6.7 months (94% CI, 4.4–7.9), 6-month PFS rate: 53.2%	[102]
GEMMK: A Phase I study of gemcitabine (gem) and pembrolizumab (pem) in patients (pts) with leiomyosarcoma (LMS) and undifferentiated pleomorphic sarcoma UPS)	1	LMS, UPS	mPFS: 5.1 months (95% CI, 2–9)	[96]
Phase II Study of Eribulin plus Pembrolizumab in Metastatic Soft-tissue Sarcomas: Clinical Outcomes and Biological Correlates	II	LMS, LPS, UPS, other	LMS ORR: 10.5%, mPFS: 11.1 weeks (90% CI, 6.5–18.7), 12-week PFS rate: 36.8% (90% CI, 22.5–60.4)	[103]
			LPS ORR: 15%, mPFS: 31.7 weeks (90% CI, 12.4—not reached), 12-week PFS rate: 69.6% (90% CI, 54.5–89.0)	
			UPS/other ORR: 17%, mPFS: 12.4 weeks (90% CI, 6.1–30.4), 12-week PFS rate: 52.6% (36.8–75.3)	
Assessment of Doxorubicin and Pembrolizumab in Patients With Advanced Anthracycline-Naive Sarcoma	1/2	LMS, DDLPS, UPS, chondrosarcoma, other	ORR: 19%, mPFS: 8.1 months (95% CI, 7.6–10.8), 12-month PFS rate: 27% (95% CI, 14–42)	[67]
Phase II Study of Pembrolizumab in Combination with Doxorubicin in Metastatic and Unresectable Soft-Tissue Sarcoma	2	LMS, DDLPS, UPS, other	ORR: 36.7% (95% CI, 19.9–56.1), mPFS: 5.7 months (95% CI, 4.1–8.9), 12-month PFS rate: 20.3% (95% CI, 6.8–38.8)	[68]
A single-arm, open-label Phase 2 trial of doxorubicin plus zalifrelimab, a CTLA-4 inhibitor, with balstilimab, a PD-1 inhibitor, in patients with advanced/metastatic soft tissue sarcomas	2	Multiple STS histologies	ORR: 36% (95% CI, 19–56), mPFS: 25.6 weeks (95%CI, 24.0–44.9), 6-month PFS rate: 52% (95%CI, 31–72)	[104]
ImmunoSarc2: A Spanish Sarcoma Group (GEIS) Phase Ib trial of doxorubicin and dacarbazine plus nivolumab in first line treatment of advanced leiomyosarcoma	1b	LMS	ORR: 56.2%, mPFS: 8.67 months (95% CI, 7.96–9.37)	[95]
Axitinib plus pembrolizumab in patients with advanced sarcomas including alveolar soft-part sarcoma: a single-centre, single-arm, Phase 2 trial		ASPS, UPS, Leiomyosarcoma, DDLPS, other	ORR: 25% (95% CI, 12.1–43.8), mPFS: 4.7 months (95% CI, 3.0 to 9.4), 6-month PFS:46.9%(95% CI, 29.2 to 62.8)	[105]
Nivolumab and sunitinib combination in advanced soft tissue sarcomas: a multicenter, single-arm, Phase Ib/II trial	1b/2	UPS, Extraskeletal myxoid chondrosarcoma ASPS, SFT, ES, CCS, angiosarcoma	ORR: 13%, mPFS: 5.6 months (95% CI, 3.0–8.1), 6-month PFS rate: 48% (95% CI, 41 to 55)	[106]
Regomune—a Phase II study of regorafenib + avelumab in solid tumours: Results of the soft tissue sarcoma (STS) cohort	2	LMS, LPS, SS, UPS, other	ORR: 9.3%, mPFS: 1.8 months (95% CI, 1.7–3.5), 6-month PFS rate: 22.1% (95% CI, 11–35.7)	[107]
Randomised Phase II trial of cabozantinib combined with PD-1 and CTLA-4 inhibition versus cabozantinib in metastatic soft tissue sarcoma	2	Multiple STS histologies	Combination arm ORR: 11%, mPFS: 5.4 months Monotherapy(cabozantinib) ORR: 6%, mPFS: 3.8 months	[108]
Durvalumab plus pazopanib combination in patients with advanced soft tissue sarcomas: a Phase II trial	2	LMS, MPNST, SS, MFS, DSRCT, UPS, DDLPS, CCS, ASPS, ESS, angiosarcoma, other	ORR: 30.4%, mPFS: 7.7 months (95% CI, 5.7–10.4)	[109]

ORR: objective response rate, mPFS: median progression-free survival, LMS: leiomyosarcoma, LPS: liposarcoma, DDLPS: dedifferentiated liposarcoma, UPS: undifferentiated pleomorphic sarcoma, SS: synovial sarcoma, ASPS: alveolar soft-part sarcoma, CCS: clear cell sarcoma, ES: epithelioid sarcoma, SFT: solitary fibrous tumour, MPNST: malignant peripheral nerve sheath tumour, MFS: myxofibrosarcoma, DSRCT: desmoplastic small round cell tumour, ESS: endometrial stromal sarcoma. The outcome refers to the overall STS population unless referring to specific histologic subtype.

## Glossary

STS	Soft tissue sarcoma
*ALK*	Anaplastic lymphoma kinase
*NTRK*	Neurotrophic Tyrosine Receptor Kinase
ER	Estrogen Receptor
PR	Progesterone Receptor
PFS	Progression-free survival
ASPS	Alveolar Soft Part Sarcoma
UPS	Undifferentiated Pleomorphic Sarcoma
MFS	Myxofibrosarcoma
PD-1	Programmed cell death protein 1
CTLA-4	Cytotoxic T-lymphocyte-associated protein 4
*KIT*	KIT Proto-Oncogene
*PDGFRA*	Platelet-derived growth factor receptor alpha
*NF1*	Neurofibromatosis 1
*BRAF*	B-Raf Proto-Oncogene, Serine/Threonine Kinase
SDH	Succinate Dehydrogenase
ATP	adenosine triphosphate
HSP90	Heat shock protein 90
HIF1a	Hypoxia-Inducible Factor 1 Alpha
HIF2a	Hypoxia-Inducible Factor 2 Alpha
DR5	death receptor 5
TRAIL	TNF-related apoptosis-inducing ligand
ITT	Intention to treat population
mPFS	median progression-free survival
mOS	median overall survival
FDA	Food and Drug Administration
PPE	palmar-plantar erythrodysesthesia
ctDNA	circulating tumour DNA
NGS	next generation sequencing
TRAEs	treatment-related adverse events
*FGF*	Fibroblast Growth Factor
PDX	Patient-derived xenograft
FGFR	FGF receptor
LPS	liposarcoma
WDLPS	Well-differentiated liposarcoma
DDLPS	dedifferentiated liposarcoma
MDM2	Mouse double minute 2 homolog
CDK4	Cyclin-dependent kinase 4
Jun	Jun Proto-Oncogene
ASK1/MAP3K5	Mitogen-Activated Protein Kinase Kinase Kinase 5
CI	confidence interval
HR	Hazard ratio
NE	not estimable
DLT	Dose-limiting toxicity
LMS	Leiomyosarcoma
XPO1	Exportin 1
*CALB1*	calbindin 1
*MLPS*	*myxoid round liposarcoma*
DDIT3	DNA damage-inducible transcript 3
FUS	fused in sarcoma
EWSR1	EWS RNA Binding Protein 1
*SS*	synovial sarcoma
SWI/SNF	Switch/Sucrose Non-Fermentable
PRC	Polycomb Repressive Complex
SMARCB1	SWI/SNF-related matrix-associated actin-dependent regulator of chromatin subfamily B member 1
INI1	Integrase Interactor 1
PRC1	Polycomb Repressive Complex 1
PRC2	Polycomb Repressive Complex 2
CTA	Cancer Testis Antigen
MAGE-A4	Melanoma-Associated Antigen-4
NY-ESO-1	New York Esophageal Squamous Cell Carcinoma-1
PRAME	Preferentially Expressed Antigen in Melanoma
HLA	leukocyte antigen
afami-cel	afamitresgene autoleucel
ORR	objective response rate
PR	partial response
SD	stable disease
CR	complete response
PD	progressive disease
PFS	progression free survival
OS	overall survival
LPA	long peptide antigen
CRS	Cytokine release syndrome
CRISPR/Cas9	Clustered regularly interspaced short palindrome repeats/Cas9
siRNA	Small interfering RNA
TP53	Tumor Protein P53
ATRX	ATRX Chromatin Remodeler
RB1	RB Transcriptional Corepressor 1
PTEN	Phosphatase And Tensin Homolog
RAD51	RAD51 Recombinase
ATR	ataxia telangiectasia and Rad3 related
UPS	Undifferentiated pleomorphic sarcoma
*CDKN2A*	cyclin-dependent kinase inhibitor 2A
NPR	non-progression rate
HNFS	head, neck, face and scalp
KDR	Kinase Insert Domain Receptor
PIK3CA	Phosphatidylinositol-4,5-Bisphosphate 3-Kinase Catalytic Subunit Alpha
Mb	Megabase
PD1	Programmed Cell Death 1
EZH2	Enhancer of zeste homolog 2
PROTACS	proteolysis targeting chimeras
TIGIT	T cell immunoreceptor with Ig and ITIM domains
mDOR	median duration of response
NTRK	neurotrophic tyrosine receptor kinase
TSC1	tuberous sclerosis 1
TSC2	tuberous sclerosis 2
mTOR	Mammalian target of rapamycin
CTNNB1	Catenin Beta 1
FAP	familial adenomatous polyposis
APC	adenomatous polyposis coli
MGD	molecular glue degraders
TAZ	Transcriptional Co-activator with a PDZ-motif
CAMTA1	Calmodulin Binding Transcription Activator 1
YAP	Yes-associated Protein
TFE3	Transcription Factor E3
EWSR1	EWS RNA Binding Protein 1
FLI1	friend leukemia virus integration 1
FOXO1	(forkhead box O1)
PAX3	(paired box 3)
PAX7	(paired box 7)
